# Physical frailty intensifies the positive association of oral frailty with poor global cognitive function and executive function among older adults especially for females: a cross-sectional study

**DOI:** 10.1186/s12877-024-05056-4

**Published:** 2024-05-29

**Authors:** Yang Fei, Shuzhen Niu, Xueru Xi, Wenping Tang, Yue Zhao, Ge Zhang, Xiaohong Yu, Cheng Li, Xinru Li, Ying Liu, Yaxin Li, Yueheng Yin, Yan Cui, Xianwen Li

**Affiliations:** 1 School of Nursing, Nanjing Medical University, 101 Longmian Avenue, Jiangning District, Nanjing, China; 2 Department of Geriatrics (Geriatric Neurology), the Affiliated Brain Hospital of Nanjing Medical University, 264 Guangzhou Road, Nanjing, China

**Keywords:** Global cognitive function, Executive function, Oral frailty, Physical frailty, Older adults

## Abstract

**Background:**

Oral frailty is reported to increase the risk of new onset of mild cognitive impairment. Whereas, the association of oral frailty with cognition among older adults in both physical frail and non-physical frail status has not been sufficiently explored, and whether there are sex differences in the association is unclear. This study investigated the association of oral frailty and physical frailty with global cognitive function and executive function among older adults, as well as the sex differences in such association.

**Methods:**

This cross-sectional study included 307 participants aged ≥ 60 years old from communities between June 2023 and August 2023, in Nanjing, China. Global cognitive function and executive function were assessed by using the Montreal Cognitive Assessment (MoCA) and Trail Making Tests A (TMT-A), respectively. Oral frailty was identified by the combination of natural tooth, Oral Frailty Index-8 (OFI-8), and oral diadochokinesis. Physical frailty was measured by using Fried phenotype model which contained 5 criteria: unintentional weight loss, weakness, exhaustion, slowness, and low physical activity. Multiple linear regression analyses for overall participants and stratified by sex and presence or absence of physical frailty were performed, respectively, to examine the association between oral frailty and cognitive functions.

**Results:**

The median age of participants was 70 years old. The study included 158 (51.5%) females, 53 (17.3%) individuals with physical frailty, and 65 (21.2%) participants with oral frailty. After adjustment, the association between oral frailty and global cognitive function was observed in the physical frailty group (B = -2.67, 95% Confidence Interval [CI]: -5.27 to -0.07, *p* = 0.045) and the females with physical frailty (B = -4, 95% CI: -7.41 to -0.58, *p* = 0.024). Oral frailty was associated with executive function in overall participants (B = 0.12, 95% CI: 0.01 to 0.22, *p* = 0.037), physical frailty group (B = 23.68, 95% CI: 1.37 to 45.99, *p* = 0.038). In the adjusted models, oral frailty was significantly associated with executive function in all females (B = 0.21, 95% CI: 0.05 to 0.36, *p* = 0.009), in females without physical frailty (B = 0.19, 95% CI: 0.02 to 0.36, *p* = 0.027), and in females with physical frailty (B = 48.69, 95% CI: 7.17 to 90.21, *p* = 0.024).

**Conclusions:**

Physical frailty intensifies the positive association of oral frailty with poor global cognitive function and executive function among older adults, particularly among females. It is ponderable to consider sex differences and facilitate the management of physical frailty when it comes to promoting cognitive health based on the perspective of oral health among older adults.

## Introduction

There are more than 55 million people suffering from dementia worldwide [1], and the long-term care and financial burden caused by dementia are substantial [2]. Mild cognitive impairment (MCI) represents a vital prodromal phase of dementia (especially Alzheimer’s disease), and more than half of MCI patients progress to dementia within 5 years [3]. Identifying and managing the modifiable risk factors of early cognitive impairment are conducive to preventing or decelerating the progression of dementia and reducing the care burden. Recently, the association between oral health and cognition has been arousing more interests in the geriatric research area.

Oral health is regarded as an essential component of healthy aging [4]. Previous studies suggested that declined oral conditions, such as tooth loss or masticatory dysfunction which decreased the sensorimotor stimulation produced by the chewing process to the brain [5], contributed to the degeneration of cognitive function. Recently, given the complexity and multidimensional nature of oral health, the concept of oral frailty is proposed which is determined by a combination of multiple oral health-related indicators [6, 7], emphasizing the age-related gradual decline of various oral conditions accompanied by deteriorative physical and mental functions [8]. A previous study had reported the association between oral frailty and the decline of global cognitive function, which indicated that oral frailty increased the risk of new onset of MCI [9].

Executive function (EF) is an important subdomain of cognitive function. Subtle EF deficits occur in the very early stage of cognitive impairment [10], which could predict the progress of cognitive impairment [11]. EF deficits jeopardize individuals’ instrumental activities of daily living [12] and increase the occurrence of disability in older adults [13]. Identifying the signs of EF deficits profoundly benefits the cognitive health of older adults. Naorungroj et al.’s [14] cross-sectional study indicated that the tooth loss and gingival bleeding were considered as markers of poorer EF among middle-aged and elderly people. Whereas Yang et al. reported that the use of dentures mitigated the adverse effects of tooth loss on cognitive impairment [15]. It implied that the single oral health indicator might be insufficient to reflect the association between oral status and EF. Consequently, oral frailty which compressively reflects oral health status might perform better as the marker of poor EF. Nevertheless, the association of oral frailty with EF among older adults has not been clarified.

Physical frailty is a complicated, multidimensional syndrome, generally referring to the decline of physiological reserve and lower tolerance for stressful events, which is regarded as the risk factor for diverse adverse health-related outcomes [16]. Previous evidence indicated oral frailty is regarded as a risk factor for physical frailty [6], and physical frailty could aggravate cognitive decline due to multiple biological, psychological, and social factors [17]. It implied that physical frailty could affect the association between oral frailty and poor cognitive function. Additionally, we also noticed that Nagatani et al. [9] found that the association between oral frailty and new-onset MCI among healthy participants was insignificant, while the risk of new-onset MCI increased when individuals exhibited physical frailty coexisted with oral frailty. It meant that compared to oral frailty alone, the coexistence of oral frailty and physical frailty might deteriorate the impairment of global cognitive function. However, the association of oral frailty with EF among older adults in the case of the presence or absence of physical frailty has not been reported yet.

**Fig. 1:**
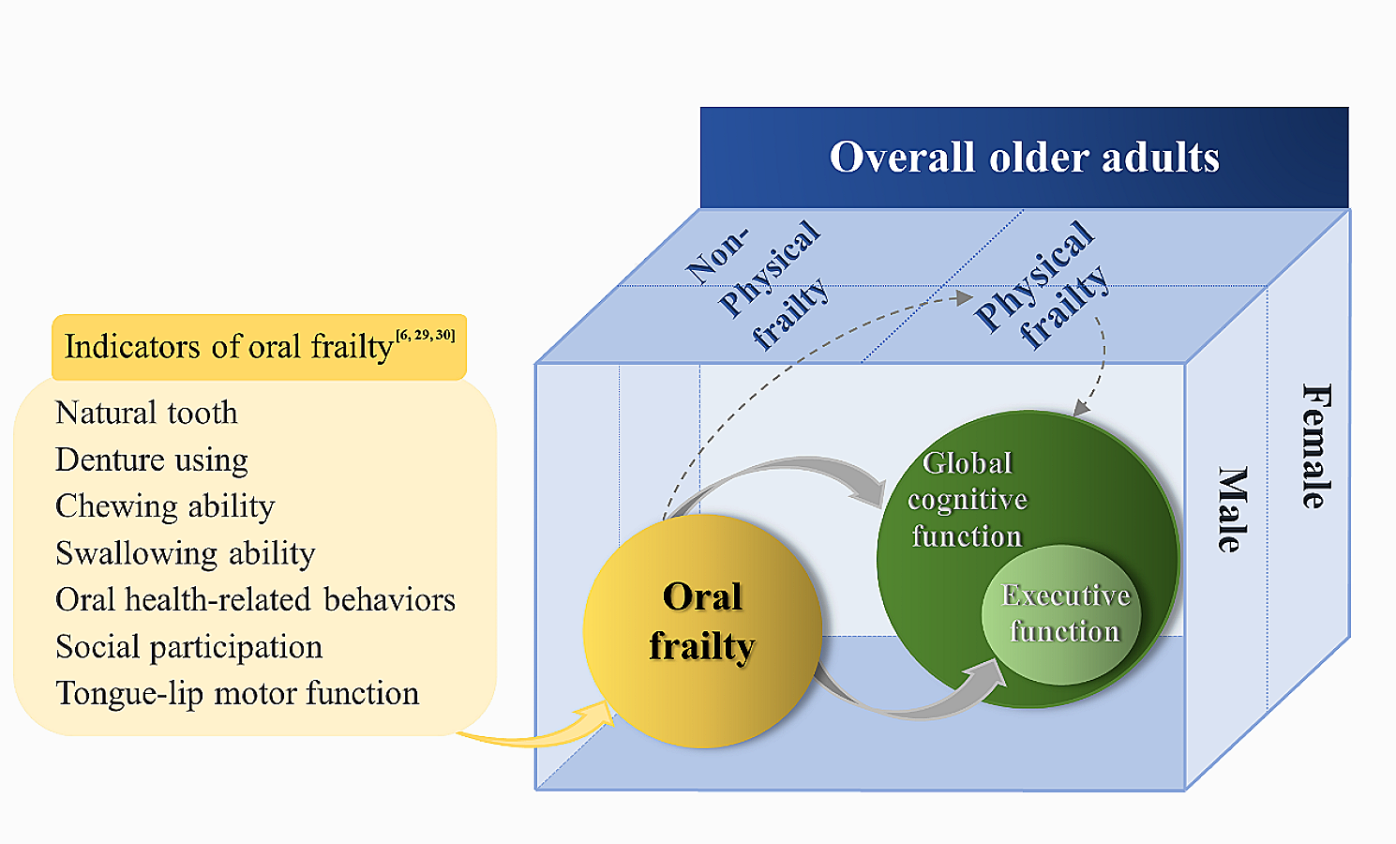
The conceptual framework of the association of oral frailty and physical frailty with cognitive function Notes: solid line arrows indicate the directionality of the association between oral frailty and poor cognitive functions. Dotted lines arrows indicate the pathway that oral frailty links to physical frailty, and physical frailty links to poor cognitive functions

Notably, it is crucial to consider the sex differences in the association between oral frailty and physical frailty with cognitive function. Females were vulnerable to poor oral status [18, 19] and physical frailty in later life [20] attributed to several factors, e.g., hormonal readiness. Additionally, studies reported that compared to males, declined oral status was robustly associated with physical frailty in females [21], while physical frailty was significantly correlated with subjective cognitive decline in females [22]. We concluded that no matter oral frailty or physical frailty, both would lead to greater adverse effects on females’ health. Therefore, we sought to explore whether there were sex differences in the association of oral frailty and physical frailty with cognitive function among older adults. Particularly, we speculated such potential associations were prominent in female older adults. Thus, this study aimed to investigate the association of oral frailty and physical frailty with global cognitive function and EF among older adults, as well as the sex differences in such association. We established the conceptual framework of the study and showed the details in Fig. 1.

## Methods

### Study design and participants

This was a cross-sectional study and conducted by adhering to the strengthening the reporting of observational studies in epidemiology statement (STROBE) [23]. The study was approved by the Ethical Committee of the Nanjing Medical University (NMU2023-562). All participants signed the informed consent before the survey. Participants aged ≥ 60 years old were recruited from 6 communities in Jiangning District, Nanjing, China, from June 2023 to August 2023 through a two-stage sampling method. People with cerebral cardiovascular disease, major brain injury, severe psychiatric or neurodegenerative disorders, severe hearing or visual problems, and serious physical dysfunction which might affect the completion of the assessment tasks, were excluded from the study. Eligible participants were invited into a quiet room in community healthcare centers to undergo a set of assessments. Face-to-face structured questionnaire interviews and physical function evaluations were conducted by professionally trained investigators.

### Measures

#### Global cognitive function

The Montreal Cognitive Assessment (MoCA) was used to measure global cognitive function, which is a 30-point test administered in 10 to 15 min, and higher scores indicate better cognition. MoCA contains 12 items for 8 cognitive domains. Details of the specific MoCA items were introduced by Nasreddine et al. [24]. In the present study, the total score of the measure was adjusted based on the education years, namely one point was added with 12 years or less of education [25].

#### Executive function

EF of participants was assessed through Trail Making Tests A (TMT-A). Individuals were instructed to draw a line as rapidly as possible joining consecutive numbers (1, 2, 3…24, 25) [26], without lifting the pen from the paper. Investigators recorded the time (seconds) spent by the participants to complete the TMT-A tests, and a longer time indicated a poor EF [27]. When an error was made, the investigator pointed it out immediately and requested the participant to correct it. The tests not completed within six minutes were stopped, while the test time and the number of errors were recorded [28].

#### Oral frailty

In the present study, oral frailty was assessed by the following combination of indicators: fewer than 20 natural teeth [29], the score of Oral Frailty Index-8 (OFI-8) ≥ 4, and the poor status of oral diadochokinesis (ODK). Participants who met these conditions were classified into the oral frailty group, while other cases were classified into the non-oral frailty group.

The OFI-8 scale is a screening questionnaire consisting of 8 items covering the aspects of tooth loss and denture using, the deterioration of general chewing ability and swallowing ability, oral health-related behaviors, and decreased social participation. The specific items are Q1. “Do you have any difficulties eating tough foods compared to 6 months ago? (Yes: 2 points)”, Q2. “Have you choked on your tea or soup recently? (Yes: 2 points)”, Q3. “Do you use dentures? (Yes: 2 points)”, Q4. “Do you often have a dry mouth? (Yes: 1 point)”, Q5. “Do you go out less frequently than you did last year? (Yes: 1 point)”, Q6. Can you eat hard foods like squid jerky or pickled radish? (No: 1 point)”, Q7. “How many times do you brush your teeth in a day? (3 or more times/day) (No: 1 point)”, and Q8. “Do you visit a dental clinic at least annually? (No: 1 point)”. The total OFI-8 score ranges from 0 to 11, with higher scores indicating poorer oral health. An OFI-8 score of ≥ 4 points means that the individuals are at high risk of new-onset oral frailty. The tool had been tested with sound sensitivity and specificity, and Cronbach’s α coefficient is 0.692 [30].

ODK is broadly used to evaluate tongue-lip motor function [31]. Participants were asked to articulate each syllable (“pa”, “ta”, “ka”) repetitively as fast as possible 20 times. Investigators used the digital counter of phone to count the time (seconds) of 20 times articulations of each syllable in a consistent standard. Before the official test, participants practiced 2 or 3 times to familiarize themselves with the test rules. We divided 20 by the total time taken to pronounce each syllable using statistical software, by which the articulation times of each syllable per second were calculated. The male whose articulation times of “pa” and “ta” were < 5.2 times/s, with “ka” < 4.4 times/s meanwhile, as well as the female whose articulation times of “pa” was < 5.6 times/s, with “ta”< 5.4 times/s and “ka” < 5.0 times/s meanwhile, were considered to have a poor status of ODK [6]. Besides, other cases were classified as non-poor status of ODK.

The measurement of oral frailty was conducted by professionally trained investigators. The inter-rater reliability of assessment for oral frailty was assessed in a pilot test. The intraclass correlation coefficient (ICC) of the OFI-8 scale, “pa” of ODK, “ta” of ODK, and “ka” of ODK were 0.978, 0.959, 0.976, and 0.935, respectively, indicating sound inter-rater reliability.

#### Physical frailty

Physical frailty was measured based on the Fried phenotype model, containing 5 criteria [32–34]. I. Unintentional weight loss: Response of “yes” to “Compared to one year ago, have you lost 3 kg or more in weight? ”. II. Weakness: Response of “yes” to “Do you need more efforts to screw on bottle caps or carry heavy objects compared to your conditions last year?” III. Exhaustion: Response of “yes” to “During the past year, have you felt that you have trouble doing everything and you get tired easily?”. IV. Low physical activity. If male older adults walk less than 2.5 h per week or female older adults walk less than 2 h per week, this was defined as “Low physical activity”. The walking time was reported by participants. V. Slowness: If a participant’s normal gait speed was < 0.75 m/s, this was defined as “slow gait speed”. Gait speed was calculated as the walk of a 6-meter distance divided by walking time (seconds). Participants meeting three or more criteria were considered as physical frailty group, and other cases were categorized into the non-physical frailty group.

### Covariates

Sociodemographic factors were age (60 ~ 69 years, 70 ~ 79 years, and ≥ 80 years), sex (female, male), education level (analphabetic, primary, secondary, and tertiary), and the average monthly income (Chinese yuan /month) (≤ 2,000, 2,000–4,000, and ≥ 4,000).

### Sample size

The sample size was estimated through PASS (Power Analysis and Sample Size) 2017 Statistical software (NCSS LLC., Kaysville, U.T., USA). The squared multiple correlation coefficient of the multiple linear regression analyses, namely ${R}^{2}$or the coefficient of determination, was used as the measure of the effect size parameter, upon which the power analysis and sample size were based. Given that the influence of the independent variable on the corresponding dependent variable was unclear, we used the unconditional (Random X’ s) model. We set a significant level of 0.05, 0.80 power of the analysis, and an estimated effect size of 0.20 (${\rho 1}^{2}$), rejecting the null hypothesis that the population effect size is ≤ 0.1 (${\rho 0}^{2}$) [35]. Considering a 10% dropout rate [36] during the survey, a total of 299 participants were needed at least.

### Statistical analysis

Statistical analysis was performed with SPSS Statistics software version 27.0 (IBM Corp., Armonk, NY, USA). A *p*-value < 0.05 indicated statistical significance. Cases with missing data were excluded in the final data analysis and outliers of variables were removed according to the box plots. Sociodemographic factors were described as frequencies (N) and percentages (%). In this study, the total scores of MoCA and the time (seconds) of TMT-A were regarded as continuous variables. Oral frailty, physical frailty, and sex were used as categorical variables. Age, education level, and average monthly income were regarded as categorical variables. Normality was assessed through the Kolmogorov-Smirnov test or Shapiro-Wilk test. Cognitive functions for different groups stratified by different variables in the overall participants and non-physical frailty group were expressed as median (interquartile range) due to skewed distribution, which were presented as the mean and standard deviation (SD) in the physical frailty group since meeting normality. Comparisons of groups stratified by different variables in the overall participants and non-physical frailty group were conducted through the Mann-Whitney U test or Kruskal-Wallis H test, while an independent sample *t-*test or one-way analysis of variance was performed in the physical frailty group according to the data distribution. Linear regression analyses among overall participants and stratified by sex and presence or absence of physical frailty were performed, respectively, to explore the association between oral frailty and cognitive functions. The dependent variable of the analysis was the score of MoCA and the taken time for the TMT-A test, while the independent variable was oral frailty. The time of the TMT-A test was included in the regression analysis after log-transformation (Logarithm with base e) in the overall participants and non-physical frailty group due to the skewed distribution of data, while the raw data was included in the analysis of the physical frailty group. Our study presented the results of unadjusted analyses and adjusted analyses. Multiple linear regression models were adjusted for the above covariates. As this study did not highlight the extent to which the different independent variables were associated with the dependent variable and the independent variables were coded into categorical variables, we did not standardize the raw data but presented the unstandardized regression coefficients B and the 95% confidence interval (CI) for B in the results. The multicollinearity between key variables was examined by the values of Tolerance and Variance Inflation Factor.

## Results

### Participants characteristics

A total of 345 participants were recruited initially, and 19 cases were deleted due to the missing data on the number of teeth, the time of the TMT-A test and ODK. Another 19 cases were deleted for the outliers of the spent time and the number of errors in the TMT-A test. Finally, the data from 307 participants was included in the statistical analysis. The age range of participants was from 60 to 90 years old, with a median age of 70 years old. There were 158 (51.5%) female, 53 (17.3%) individuals with physical frailty, 65 (21.2%) participants with oral frailty participating in the study. Table 1 presents characteristics of participants and Table 2 shows the results of the comparisons of cognitive functions between groups stratified by sociodemographic factors, the presence or absence of oral frailty and physical frailty. Overall, there were statistically significant differences in cognitive functions between individuals of different sociodemographic backgrounds.

**Table 1:** Sociodemographic characteristics of participants (*N* = 307)

	Overall participants (*N* = 307) *n* (%)	Non-Physical frailty group (*N* = 254) *n* (%)	Physical frailty group (*N* = 53) *n* (%)	Non-Oral frailty group (*N* = 242) *n* (%)	Oral frailty group (*N* = 65) *n* (%)
**Age (years)**					
60–69	136 (44.3)	120 (47.2)	16 (30.2)	117 (48.3)	19(29.2)
70–79	152 (49.5)	120 (47.2)	32 (60.4)	114 (47.1)	38(58.5)
≥ 80	19 (6.2)	14 (5.5)	5 (9.4)	11 (4.5)	8(12.3)
**Sex**					
Female	158 (51.5)	134 (52.8)	24 (45.3)	126 (52.1)	32(49.2)
Male	149 (48.5)	120 (47.2)	29 (54.7)	116 (47.9)	33(50.8)
**Education level**					
Analphabetic	59 (19.2)	45 (17.7)	14 (26.4)	46 (19.0)	13 (20.0)
Primary	137 (44.6)	114 (44.9)	23 (43.4)	105 (43.4)	32 (49.2)
Secondary	70 (22.8)	60 (23.6)	10 (18.9)	58 (24.0)	12 (18.5)
Tertiary	41 (13.4)	35 (13.8)	6 (11.3)	33 (13.6)	8 (12.3)
**The average monthly income (Chinese yuan / Renminbi (RMB))**					
≤ 2000	207 (67.4)	166 (65.4)	41 (77.4)	158 (65.3)	49 (75.4)
2000-4000	54 (17.6)	50 (19.7)	4 (7.5)	45 (18.6)	9 (13.8)
≥ 4000	46 (15)	38 (15)	8 (15.1)	39 (16.1)	7 (10.8)
**Oral frailty**					
Non-Oral frailty	242 (78.8)	201 (79.1)	41 (77.4)		
Oral frailty	65 (21.2)	53 (20.9)	12 (22.6)		
**Physical frailty**					
Non-Physical frailty	254 (82.7)			201 (83.1)	53 (81.5)
Physical frailty	53 (17.3)			41 (16.9)	12 (18.5)

**Table 2:** Comparisons of cognitive functions between groups stratified by sociodemographic characteristics among older adults

Variables	Overall participants (*N* = 307)					Non-Physical frailty (*N* = 254)					Physical frailty (*N* = 53)				
	GCF^a^	*p*	EF^b^	*p*		GCF^a^	*p*	EF^b^	*p*		GCF^a^	*p*	EF^b^	*p*	
**Age (years)**															
60–69	21 (17, 23)	0.007	74 (54, 101.5)	< 0.001		21 (18, 23)	< 0.001	69.5 (53, 100)	< 0.001		17.69 ± 4.80	0.689	95.63 ± 31.80	0.935	
70–79	20 (17, 22)		90 (66.5, 119)			20 (17, 22)		87.5 (68.25, 118.5)			18.41 ± 4.35		99.5 ± 39.81		
≥ 80	17 (15, 19)		106 (84, 164)			17 (14.75, 18)		108 (83, 177.75)			19.6 ± 3.78		100.2 ± 19.39		
**Sex**															
Female	19 (16, 22)	< 0.001	93 (62.5, 120)	0.005		19 (16, 22)	< 0.001	89.5 (61, 119)	0.002		17.63 ± 4.13	0.312	101.63 ± 39.61	0.553	
Male	21 (18, 23)		80 (57, 101.5)			22 (18.25, 24)		75.5 (55.25, 99.75)			18.86 ± 4.60		95.72 ± 32.43		
**Education level**															
Analphabetic	16 (14, 18)	< 0.001	110 (89, 140)	< 0.001		16 (14, 20)	< 0.001	109 (89.5, 142.5)	< 0.001		15.57 ± 2.90	0.002	113.79 ± 41.04	0.022	
Primary	19 (17, 22)		89 (69, 117.5)			20 (17, 22)		87.5 (67.5, 109.3)			17.91 ± 4.71		103.65 ± 34.97		
Secondary	22 (20, 24)		65 (51, 90.25)			23 (20.25, 24.75)		64.5 (50.25, 81.5)			20.6 ± 2.46		82.3 ± 22.03		
Tertiary	23 (21, 25)		64 (49.5, 82)			23 (21, 25)		62 (47, 81)			22.33 ± 4.32		69.17 ± 15.21		
The average monthly income (Chinese yuan / RMB*)															
≤ 2000	19 (16, 22)	< 0.001	91 (66, 120)	< 0.001		20 (16.75, 22)	0.002	90 (64.75, 119)	< 0.001		17.73 ± 4.37	0.22	102.73 ± 36.57	0.266	
2000-4000	22 (19.75, 23)		65 (52.5, 81)			22 (20, 23.25)		63 (51, 81)			20.25 ± 1.5		83 ± 18.11		
≥ 4000	21 (17.75, 24)		79.5 (57.5, 103.5)			21 (17.75, 24)		82 (57.5, 103.5)			20.25 ± 5.01		83.88 ± 34.0		
**Oral Frailty**															
Non-Oral frailty	20.5 (17, 23)	0.081	81 (58, 105)	0.009		21 (17.5, 23)	0.234	80 (58, 104.5)	0.018		18.76 ± 4.43	0.166	93.51 ± 29.7	0.170	
Oral frailty	19 (16, 22.5)		97 (69.5, 131)			20 (17, 23)		97 (67.5, 128.5)			16.75 ± 4.05		115.08 ± 48.97		
**Physical Frailty**															
Non-Physical frailty	20.5 (17, 23)	0.01	81 (58, 109.25)	0.055											
Physical frailty	18 (15, 22)		91 (73, 124)												

**Notes**: GCF^a^: Global Cognitive Function; EF^b^: Executive Function; RMB*: Renminbi

Cognitive functions for different groups stratified by different variables in the overall participants and non-physical frailty group were expressed as median (interquartile range) due to skewed distribution, which were presented as mean ± standard deviation (SD) in the physical frailty group since meeting normality

*P* value < 0.05 is statistically significant. *P* values in the overall participants and non-physical frailty group were calculated through the Mann-Whitney U test (two-category data) or Kruskal-Wallis H test (ordinal categorical variables), and *P* values in the physical frailty group were derived based on an independent sample t-test (two-category data) or one-way analysis of variance (ordinal categorical variables)

### The association of oral frailty and physical frailty with cognitive function

The results showed that the participants with only oral frailty had poor EF, and those diagnosed with both oral frailty and physical frailty had poor EF and global cognitive function. For the EF, we observed a significant association between oral frailty and EF in the crude model of the overall participants (B = 0.17, 95% CI: 0.05 to 0.29, *p* = 0.006), which was still significant (B = 0.12, 95% CI: 0.01 to 0.22, *p* = 0.037) after adjustment for the above covariates. There was also a relationship of that for the crude model of non-physical frailty group (B = 0.17, 95% CI: 0.03 to 0.3, *p* = 0.016), however, this was not significant after adjustment (B = 0.1, 95% CI: -0.02 to 0.22, *p* = 0.113). Inversely, the association was significant in the adjusted model (B = 23.68, 95% CI: 1.37 to 45.99, *p* = 0.038) instead of the crude model for the physical frailty group (B = 21.57, 95% CI: -1.34 to 44.48, *p* = 0.064). For global cognitive function, although oral frailty was not related to global cognitive function either in the overall participants or non-physical frailty group, there was a significant association in the adjusted model of the physical frailty group (B = -2.67, 95% CI: -5.27 to -0.07, *p* = 0.045).

### The sex difference in the association of oral frailty and physical frailty with cognitive function

The results showed that the positive association of oral frailty and physical frailty with cognitive function was significant in females, however, it was not significant among males. There was no significant difference in EF or global cognitive function between males with or without oral frailty. In contrast, females with oral frailty had poor EF compared to those without oral frailty. Besides, Females with both oral frailty and physical frailty also had poor global cognitive function compared to females with only oral frailty. For EF, in the adjusted models, oral frailty was significantly associated with EF in all females (B = 0.21, 95% CI: 0.05 to 0.36, *p* = 0.009), in females without physical frailty (B = 0.19, 95% CI: 0.02 to 0.36, *p* = 0.027), and in females with physical frailty (B = 48.69, 95% CI: 7.17 to 90.21, *p* = 0.024). For global cognitive function, despite the association between oral frailty and global cognitive function was not significant for the females of the overall participants and non-physical frailty group, we still observed a significant relationship of that in the adjusted model among the physical frailty group (B = -4, 95% CI:-7.41 to -0.58, *p* = 0.024). Tables 3 and 4 show the results of unadjusted and adjusted analyses of the association of oral frailty and physical frailty with global cognitive function or EF, respectively. For the results of model fitting, all adjusted models of the overall participants and females of the non-physical frailty group presented the R^2^ > 0.2 with *p*-value < 0.05. The crude model of the association between oral frailty and EF in females with physical frailty was statistically significant (R^2^ = 0.17, F = 4.40, *p* = 0.048), but the *p*-value of the adjusted model was > 0.05 (R^2^ = 0.37, F = 2.84, *p* = 0.053). We need to mention that although it was difficult to convert MoCA scores to a normal distribution, the linear regression model had acceptable residual independence, normality, and homogeneity of variance. Additionally, the results of multicollinearity were acceptable.

**Table 3:** The association of oral frailty and physical frailty with global cognitive function among older adults

Independent variables	Overall participants						Female group						Male group					
	Crude model			Adjusted model			Crude model			Adjusted model			Crude model			Adjusted model		
	B (95% CI)^a^	*p*-value		B (95% CI)^a^	*p*-value		B (95% CI)^a^	*p*-value		B (95% CI)^a^	*p*-value		B (95% CI)^a^	*p*-value		B (95% CI)^a^	*p*-value	
**Overall participants**																		
Age by group				-0.39 (-1.12, 0.34)	0.297					0.11 (-0.96, 1.18)	0.835					-0.77 (-1.79, 0.25)	0.138	
Education level				2 (1.52, 2.48)	< 0.001					2.26 (1.57, 2.94)	< 0.001					1.85 (1.15, 2.55)	< 0.001	
Average monthly income				0.23 (-0.35, 0.81)	0.434					0.54 (-0.33, 1.41)	0.223					-0.03 (-0.83, 0.78)	0.948	
Oral frailty (yes)	-1.06 (-2.23, 0.11)	0.074		-0.76 (-1.78, 0.26)	0.142		-1.49 (-3.13, 0.16)	0.077		-0.64 (-2.1, 0.83)	0.393		-0.75 (-2.34, 0.84)	0.350		-0.64 (-2.09, 0.81)	0.382	
Physical frailty (yes)				-1.29 (-2.37, -0.2)	0.021					-0.68 (-2.3, 0.94)	0.411					-1.83 (-3.33, -0.33)	0.017	
Sex (female)				1.42 (0.56, 2.27)	0.001													
**Non-Physical frailty group**																		
Age by group				-0.73 (-1.53, 0.08)	0.076					-0.09 (-1.28, 1.09)	0.876					-1.34 (-2.44, -0.25)	0.017	
Education level				1.91 (1.39, 2.44)	< 0.001					2.19 (1.39, 2.99)	< 0.001					1.71 (1, 2.42)	< 0.001	
Average monthly income				0.23 (-0.4, 0.86)	0.475					0.43 (-0.56, 1.43)	0.388					0.1 (-0.7, 0.9)	0.805	
Oral frailty (yes)	-0.82 (-2.1, 0.45)	0.204		-0.34 (-1.45, 0.77)	0.546		-1.2 (-2.98, 0.58)	0.185		-0.16 (-1.78, 1.46)	0.843		-0.4 (-2.11, 1.31)	0.644		-0.27 (-1.83, 1.28)	0.727	
Sex (female)				1.72 (0.79, 2.64)	< 0.001													
**Physical frailty group**																		
Age by group				1.16 (-0.69, 3.02)	0.214					1.48 (-1.06, 4.02)	0.237					1.27 (-1.5, 4.04)	0.354	
Education level				2.46 (1.15, 3.77)	< 0.001					2.75 (1.46, 4.03)	< 0.001					2.99 (0, 5.99)	0.050	
Average monthly income				-0.18 (-1.82, 1.47)	0.829					0.7 (-0.99, 2.39)	0.395					-1.72 (-5.38, 1.95)	0.343	
Oral frailty (yes)	-2.01 (-4.87, 0.86)	0.166		-2.67 (-5.27, -0.07)	0.045		-3.75 (-8.25, 0.75)	0.098		-4 (-7.41, -0.58)	0.024		-1.36 (-5.32, 2.59)	0.485		-1.83 (-5.73, 2.07)	0.343	
Sex (female)				-0.1 (-2.43, 2.23)	0.934													

**Notes**: In all models, the dependent variable was global cognitive function. The associations between constants and dependent variables of all linear regression analyses were significant which were not shown in the table. B (95% CI)^a^: Unstandardized regression coefficient B and 95% confidence interval of coefficient B

The adjustment variables were “Age by group”, “Education level”, “Average monthly income”, “Physical frailty” (“Physical frailty” did not serve as an adjustment variable in the non-physical or physical frailty group.), and “Sex” (“Sex” did not serve as an adjustment variable in female or male group)

**Table 4:** The association of oral frailty and physical frailty with executive function among older adults

Independent variables	Overall participants						Female group						Male group					
	Crude model			Adjusted model			Crude model			Adjusted model			Crude model			Adjusted model		
	B (95% CI)^a^	*p*-value		B (95% CI)^a^	*p*-value		B (95% CI)^a^	*p*-value		B (95% CI)^a^	*p*-value		B (95% CI)^a^	*p*-value		B (95% CI)^a^	*p*-value	
**Overall participants**																		
Age by group				0.14 (0.06, 0.22)	0.001					0.07 (-0.04, 0.19)	0.193					0.2 (0.09, 0.31)	< 0.001	
Education level				-0.14 (-0.2, -0.09)	< 0.001					-0.17 (-0.25, -0.1)	< 0.001					-0.11 (-0.19, -0.04)	0.004	
Average monthly income				-0.03 (-0.09, 0.03)	0.330					-0.04 (-0.13, 0.05)	0.366					-0.02 (-0.11, 0.06)	0.618	
Oral frailty (yes)	0.17 (0.05, 0.29)	0.006		0.12 (0.01, 0.22)	0.037		0.29 (0.12, 0.45)	< 0.001		0.21 (0.05, 0.36)	0.009		0.06 (-0.11, 0.22)	0.508		0 (-0.16, 0.15)	0.959	
Physical frailty (yes)				0.06 (-0.06, 0.18)	0.309					-0.01 (-0.18, 0.16)	0.941					0.12 (-0.04, 0.28)	0.136	
Sex (female)				-0.13 (-0.22, -0.04)	0.005													
**Non-Physical frailty group**																		
Age by group				0.16 (0.08, 0.25)	< 0.001					0.08 (-0.05, 0.2)	0.225					0.27 (0.14, 0.39)	< 0.001	
Education level				-0.14 (-0.2, -0.08)	< 0.001					-0.17 (-0.26, -0.09)	< 0.001					-0.1 (-0.18, -0.02)	0.017	
Average monthly income				-0.03 (-0.1, 0.04)	0.401					-0.07 (-0.17, 0.04)	0.199					-0.01 (-0.1, 0.09)	0.899	
Oral frailty (yes)	0.17 (0.03, 0.3)	0.016		0.1 (-0.02, 0.22)	0.113		0.29 (0.11, 0.47)	0.002		0.19 (0.02, 0.36)	0.027		0.02 (-0.17, 0.22)	0.807		-0.05 (-0.23, 0.14)	0.625	
Sex (female)				-0.16 (-0.26, -0.06)	0.002													
**Physical frailty group**																		
Age by group				0.35 (-15.56, 16.27)	0.964					6.45 (-24.39, 37.29)	0.666					-0.04 (-19.38, 19.3)	0.997	
Education level				-16.17 (-27.41, -4.94)	0.006					-17.94 (-33.56, -2.32)	0.027					-10.02 (-30.88, 10.85)	0.332	
Average monthly income				0.26 (-13.84, 14.36)	0.971					8.21 (-12.34, 28.75)	0.413					-9.38 (-34.91, 16.16)	0.456	
Oral frailty (yes)	21.57 (-1.34, 44.48)	0.064		23.68 (1.37, 45.99)	0.038		42.45 (0.45, 84.45)	0.048		48.69 (7.17, 90.21)	0.024		11.6 (-16.18, 39.38)	0.399		10.77 (-16.42, 37.96)	0.422	
Sex (female)				-0.4 (-20.39, 19.59)	0.968													

**Notes**: In all models, the dependent variable was executive function. The associations between constants and dependent variables of all linear regression analyses were significant which were not shown in the table. B (95% CI)^a^: Unstandardized regression coefficient B and 95% confidence interval of coefficient B

The adjustment variables were “Age by group”, “Education level”, “Average monthly income”, “Physical frailty” (“Physical frailty” did not serve as an adjustment variable in the non-physical or physical frailty group.), and “Sex” (“Sex” did not serve as an adjustment variable in female or male group)

## Discussion

This study investigated the associations of oral frailty and physical frailty with cognitive functions and the sex difference of these associations using the data from community-dwelling older adults. The main findings indicated that generally, oral frailty was positively correlated with poor cognitive function, and the association was intensified by the presence of physical frailty. In addition, the associations of oral frailty and physical frailty with poor cognitive functions were prominent among females compared to that in males.

The present study demonstrated the association between oral frailty and cognitive functions among older adults. Consistent with previous study which reported that poor oral conditions constituted hazard factors of cognitive impairment [37], overall, the association of declined oral status defined by multiple indicators with poor cognitive functions was observed in the present study. Specifically, our findings indicated that oral frailty was positively associated with poor EF among overall older adults. However, oral frailty was not associated with global cognitive function among all participants in this study. It may be related to the phenomenon that mild EF deficits often occur in preclinical stage of cognitive impairment [11]. Previously, study reported EF could predict the validity of subjective memory complaints during periods while objectively measured clinical symptoms of cognitive impairment were not obvious [38], while Farias et al. reported deficits in EF were one of the strongest predictors of diagnostic conversion to MCI among older adults with normal cognition [39]. Empirical studies hint that EF is seemly vulnerable to detrimental conditions compared to global cognitive function. Our study indicated that oral frailty was more likely to impact EF compared to global cognitive function. It appeared to be reasonable to consider oral frailty as one of the early potential markers for the decline of EF among older adults.

Our study suggested that the presence of physical frailty exhibited an additive effect on the association between oral frailty and poor cognitive function. Particularly, oral frailty existing alone was only associated with EF, whereas oral frailty was positively related to both poor EF and global cognitive function when oral frailty coexisted with physical frailty. Previous studies have confirmed the detrimental effect of physical frailty on cognition. Chu et al. emphasized [40] that physical frailty was associated with lower levels and steeper declines in several cognitive domains, particularly EF. A previous study [41] found that non-demented older adults with physical frailty were associated with a decline in MoCA scores compared with those without physical frailty in the next two years. Furthermore, physical frailty and cognitive decline were considered to share some common mechanisms, including inflammation, oxidative stress, etc [42], which cause more unfavorable conditions to each other. Thus, physical frailty could further exacerbate the vulnerability of cognition. Combined with our study, in the overall participants, people with physical frailty showed a poorer global cognitive function compared to those without physical frailty, while there was no significant difference in EF in physical frailty and non-physical frailty group, and the association of oral frailty with global cognitive function or EF indeed differed in the case of the presence or absence of physical frailty. Therefore, the presence of physical frailty may imply that both global cognitive function and the specific cognitive domain of the individual had been impaired to a certain extent. Likewise, it meant that oral frailty was more likely to affect both EF and global cognitive function of individual with physical frailty. Since the process of physical frailty is modifiable or reversible [43], it indicates that regarding preventing cognitive decline through oral health intervention, clinicians should attach importance to the early screening and management of physical frailty.

The sex differences in the association of oral frailty and physical frailty with cognitive function were demonstrated in this study. Of note, no matter the presence or absence of physical frailty, oral frailty was not related to cognitive functions in males, while oral frailty was always associated with EF in females. Additionally, in the overall participants and non-physical frailty group of our study, global cognitive function and EF of females were poorer than males. A range of factors concerning society, psychology, and physiology, provided plausible explanations for the sex differences in cognition. Prior evidences suggested that compared to males, females were more likely to be less socially engaged [44], exhibit poorer mental health [45], and even experience higher exposure to pain, such as migraine [46, 47]. Besides, greater fluctuation of sex hormones for female older adults in later life than males of the same age might provide another potential explanation for the poor EF of females than males [48, 49]. Previous evidence depicted the sex differences in cognitive vulnerability. Namely, our findings implied that the cognition of females might be more susceptible to the negative effect of oral frailty. Consequently, the association of oral frailty with EF was robust in females. Likewise, physical frailty also presented an additive effect, besides EF, the association of oral frailty with global cognitive function also emerged among female older adults coexisted with oral frailty and physical frailty, which was not found in males. It appeared that the coexistence of oral frailty and physical frailty might be an important predictor of cognitive decline in females. Developing sex-specific policies or measures is essential for preventing or managing oral and cognitive health.

There were some limitations of this study. The methods for the determination of oral frailty varied greatly without a consensus, and we incorporated some generally accepted criteria to measure oral frailty in our study. However, it is important to reach a consensus to enable comparison with other research in further studies. Additionally, the participants were only recruited from Nanjing City, thus the findings of the present study might not be generalizable to the broader population of older adults. Finally, the specific causality could not be determined through our cross-sectional design, which needs to be further verified by more longitudinal and larger-scale studies.

## Conclusions

The present study indicates that physical frailty intensifies the positive association of oral frailty with poor global cognitive function and EF among older adults, particularly among females. It is ponderable to consider sex differences and facilitate the management of physical frailty when it comes to promoting cognitive health based on the perspective of oral health among older adults. Longitudinal studies with large-scale are needed to further verify the findings.

## Data Availability

The datasets used during the current study are available from the corresponding author on reasonable request.
